# Light Dependent Changes in Adenylate Methylation of the Promoter of the Mitochondrial Citrate Synthase Gene in Maize (*Zea mays* L.) Leaves

**DOI:** 10.3390/ijms232113495

**Published:** 2022-11-04

**Authors:** Alexander T. Eprintsev, Dmitry N. Fedorin, Abir U. Igamberdiev

**Affiliations:** 1 Department of Biochemistry and Cell Physiology, Voronezh State University, 394018 Voronezh, Russia; 2 Department of Biology, Memorial University of Newfoundland, St. John’s, NL A1C 5S7, Canada

**Keywords:** DNA methylation, adenine, citrate synthase, cytosine, restriction, promoter, phytochrome B, tricarboxylic acid cycle

## Abstract

Limited methyl-specific restriction of genomic DNA by endonuclease MAL1 revealed the changes in its methyl status caused by adenine modification in maize (*Zea mays* L.) leaves under different light conditions (dark, light, irradiation by red and far-red light). Incubation in the light and irradiation by red light exhibited an activating effect on DNA adenine methylase activity, which was reflected in an increase in the number of methylated adenines in GATC sites. Far-red light and darkness exhibited an opposite effect. The use of nitrite conversion of DNA followed by methyladenine-dependent restriction by MboI nuclease revealed a phytochrome B-dependent mechanism of regulation of the methyl status of adenine in the GATC sites in the promoter of the gene encoding the mitochondrial isoform of citrate synthase. Irradiation of plants with red light caused changes in the adenine methyl status of the analyzed amplicon, as evidenced by the presence of restriction products of 290, 254, and 121 nucleotides. Adenine methylation occurred at all three GATC sites in the analyzed DNA sequence. It is concluded that adenylate methylation is controlled by phytochrome B via the transcription factor PIF4 and represents an important mechanism for the tricarboxylic acid cycle regulation by light.

## 1. Introduction

Research in biochemistry and molecular biology has shown that, along with the four classic bases (adenine, guanine, cytosine and thymine), additional methylated bases exist in DNA. The base 5-Methyl-2′-deoxycytosine (^5m^C) was first discovered as a minor base in various DNAs, and *N*^6^-methyl-2′deoxyadenine (^6m^A) was subsequently found in bacterial DNA [1,2,3]. The origin of these bases in DNA was unknown until, in 1963, specific DNA methyltransferase enzymes were discovered in bacteria, and then in eukaryotes [2,4]. These enzymes transfer methyl groups from adenosyl-L-methionine selectively to certain cytosines or adenines in DNA strands. To date, adenylate methylation is recognized as one of the essential epigenetic mechanisms of adaptation [1,2]; however, its potentially important role in plants in relation to their photosynthetic performance and to adaptation to light conditions has not yet been studied.

The discovery of the DNA methylase enzymes revealed that ^5m^C and ^6m^A are not inserted in DNA directly but appear as a result of enzymatic methylation of the common bases (C or, respectively, A) in DNA strands [5,6]. The specificity, as well as functional role, of DNA methylation was unknown for a long time. Moreover, there was a widely assumed notion that these minor bases have no significant importance either in the structure of the DNA itself or in its functioning [7,8,9]. The role of DNA methylation in regulating the expression of genes encoding enzymes of energy metabolism and, in particular, of the tricarboxylic acid (TCA) cycle enzymes, has attracted attention only recently [10,11]. The effect of DNA methylation on the regulation of genes with CpG islands in the promoter is achieved due to chromatin condensation, which prevents the binding of transcription factors [12,13].

The ^6m^A level ranges from −0.0001 to 0.0003% of adenines in plants and mammals, which is several orders of magnitude lower than the ^5m^C level [14,15]. In contrast to the well-characterized RNA-^6m^A, relatively little is known about the functional importance of DNA-^6m^A in metazoan genomes and whether ^6m^A plays an important role in the dynamic regulation of biological processes [1,16,17]. It has been suggested that ^6m^A levels in eukaryotic organisms may be particularly sensitive to stress factors and even to minor changes in the environment [18,19,20].

Only recently, it became evident that DNA adenine methylation plays an important role in epigenetic regulation of vital processes in plants [21,22,23]. In particular, the evidence has been obtained that DNA methylation of adenine is essential in developmental processes and in stress response of rice [24,25], in the transition from vegetative to reproductive growth in Arabidopsis [20], and in fruit development of sea buckthorn [26]. It is becoming evident that ^6m^A is complementary to ^5m^C as a gene expression epigenomic mark in eukaryotes, carrying essential biophysical properties that affect the stability of nucleic acids and their binding affinity with other molecules, thus providing the changes in gene expression, DNA replication, and repair mechanisms [22,25].

The expression of genes encoding various enzymes of photosynthetic and respiratory metabolism is regulated by light via the phytochrome and cryptochrome systems [27,28], which imposes limitations on the operation of the TCA cycle and its entry enzyme citrate synthase [29]. The transmission of signals via the phytochrome system includes cytosine methylation of promoters of the genes of NAD-malic enzyme [30], aconitase [31], and citrate synthase [29]. Succinate dehydrogenase and fumarase represent another point of control of the TCA cycle [32,33], taking place at the transcriptional level via promoter methylation [11] and at the posttranslational level via thioredoxin [34].

The purpose of this study was to investigate the changes in the adenylate methyl status of DNA in maize leaves under different light conditions. In particular, we aimed to determine the possible role of DNA adenine methylation in the light-dependent regulation of the TCA cycle. We demonstrate that the TCA cycle is regulated by light via adenylate methylation of the promoter of the gene encoding the mitochondrial form of citrate synthase, and this process is mediated by phytochrome B via the phytochrome interacting factor PIF4.

## 2. Results

### 2.1. Methyl Status of Maize DNA

**Figure 1:**
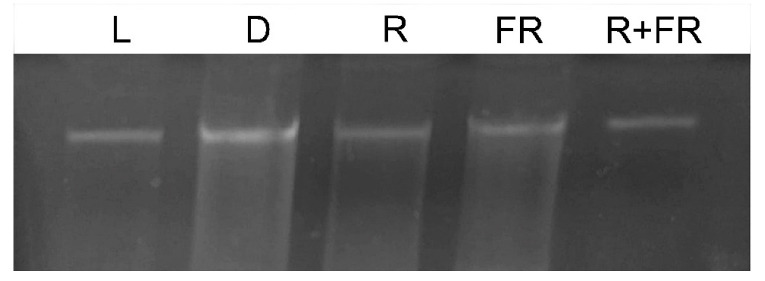
Genomic DNA from maize leaves under different light conditions. L, plants illuminated with white light; D, plants kept in darkness; R, plants illuminated with red (660 nm) light; FR, plants illuminated with far-red (730 nm) light; R+FR, plants illuminated with red (660 nm) followed by far-red (730 nm) light.

To assess the methyl status of the genomic DNA of maize leaves under different light conditions, genomic DNA was isolated using the guanidine-isothiocyanate extraction method. The use of this substance made it possible to isolate the total DNA of the cell with virtually no signs of degradation (Figure 1). To analyze the methyl status of adenine in DNA, the restriction analysis method was applied using the MAL1 endonuclease. This restriction enzyme recognizes methylated adenine in the GATC sequence and performs symmetrical restriction at this G^m6^A^TC site [35].

**Figure 2:**
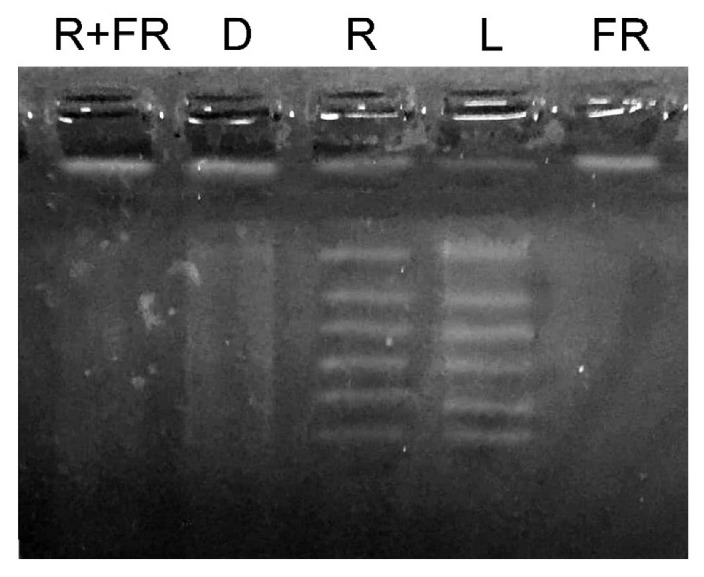
Methyl-specific restriction of genomic DNA from maize leaves under different light conditions. L, plants illuminated with white light; D, plants kept in darkness; R, plants illuminated with red (660 nm) light; FR, plants illuminated with far-red (730 nm) light; R+FR, plants illuminated with red (660 nm) followed by far-red (730 nm) light.

The results of the study of changes in the total methyl status of DNA from maize leaves under different light conditions are presented in Figure 2. Under white light and upon irradiation with red light, an increase in the genomic DNA molecule fragmentation was observed after treatment with a restriction enzyme specific for adenylate methylation (MAL1). An opposite result was observed when genomic DNA was exposed to the restriction enzyme MAL1 in the darkness, and upon irradiation by far-red light after darkness or after the exposure to red light. These treatments resulted in the insignificant or zero level of DNA fragmentation, which indicates a considerably lower number of methylated restriction sites for the MAL1 endonuclease.

### 2.2. PIF4 Expression and DNA Adenine Methylase Activity Depending on Light Regime

**Figure 3:**
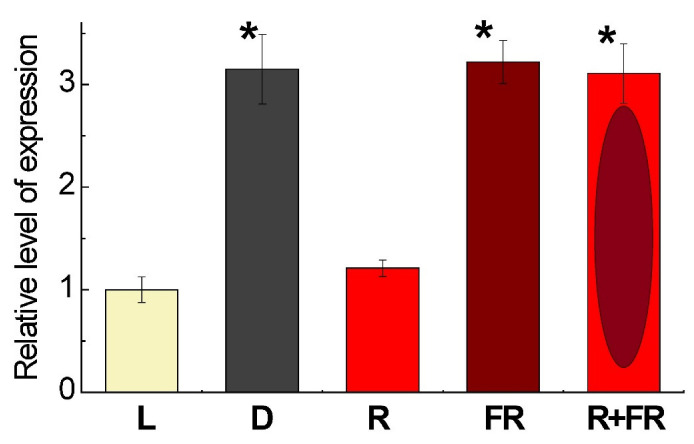
Relative level of *Pif4* gene expression in maize leaves under different light conditions. Data are the means of three biological repeats ± SD. Asterisks indicate significant differences at *p* < 0.05. Abbreviations: L, plants illuminated with white light; D, plants kept in darkness; R, plants illuminated with red (660 nm) light; FR, plants illuminated with far-red (730 nm) light; R+FR, plants illuminated with red (660 nm) followed by far-red (730 nm) light.

The level of expression of the gene *Pif4* in maize leaves depending on light conditions is presented on Figure 3. The exposure of plants on white light or their irradiation with red (660 nm) light promoting the formation of an active form of phytochrome revealed low levels of the transcripts of *Pif4*, which is the phytochrome interacting factor exhibiting the affinity to phytochrome B [36]. The incubation of plants in darkness or exposure to far-red (730 nm) light after darkness or after red light increased the levels of transcripts by threefold as compared to the plants illuminated by white or red light.

**Figure 4:**
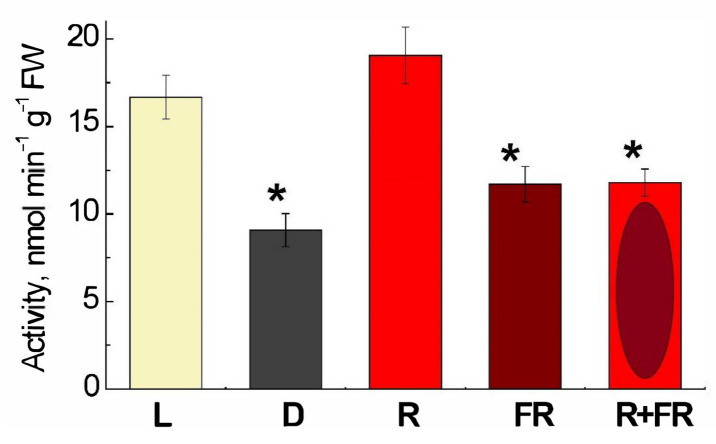
Changes in the activity of DNA adenine methylase in maize leaves under different lighting conditions. Data are the means of three biological repeats ± SD. Asterisks indicate significant differences at *p* < 0.05. Abbreviations: L, plants illuminated with white light; D, plants kept in darkness; R, plants illuminated with red (660 nm) light; FR, plants illuminated with far-red (730 nm) light; R+FR, plants illuminated with red (660 nm) followed by far-red (730 nm) light.

The DNA adenine methyltransferase (Dam methylase) activity in maize leaves upon illumination with the light of different wavelengths is demonstrated in Figure 4. Incubation of plants under white light or their irradiation with red (660 nm) light promoting the formation of an active form of phytochrome led to a twofold increase in activity, as compared to darkness. The irradiation by far-red (730 nm) light after darkness or after red light resulted in the values of activity only slightly higher than in darkness.

### 2.3. Adenylate Methyl Status of GATC Sites in the Promoter of Citrate Synthase

**Figure 5:**
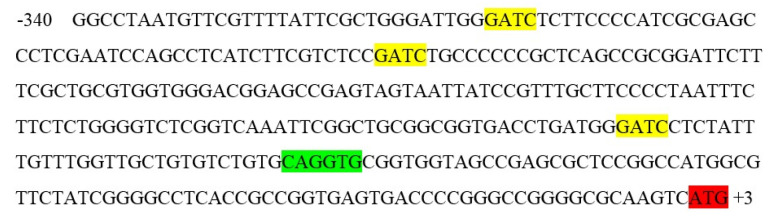
Nucleotide sequence of the amplicon of the *Csy1* gene promoter indicating potential methylation sites for DNA adenine methylase (highlighted in yellow). Green color indicates the presence of E-box. The start codon is marked in red.

To study the influence of light regime of plants on the change in the adenylate methyl status of individual GATC sites in the genomic DNA, we analyzed the promoter of the gene of the mitochondrial citrate synthase (CS, EC 2.3.3.1) isoenzyme. Previously, the mechanism of light regulation of citrate synthase was established, which includes a change in the degree of promoter methylation for cytosine in CG dinucleotides, which leads to the inhibition of *Csy1* gene expression [29]. Analysis of the nucleotide sequence of the promoter of this maize gene showed that in the region of the start codon there are three potential adenine methylation sites characteristic for Dam methylase (Figure 5).

When sodium nitrite is used under acidic conditions, chemoselective deamination of non-methylated adenines easily occurs without competing deamination of *N*^6^-adenine sites. Deamination of adenines leads to the formation of hypoxanthine bases recognized by polymerases and reverse transcriptases as guanine, while the methylated adenine sites are not deaminated and recognized as adenine. This approach, combined with high-throughput DNA sequencing, mutational analysis, and other approaches, makes it possible to identify *N*^6^-adenine sites in RNA and DNA in various sequence contexts [37].

**Figure 6:**
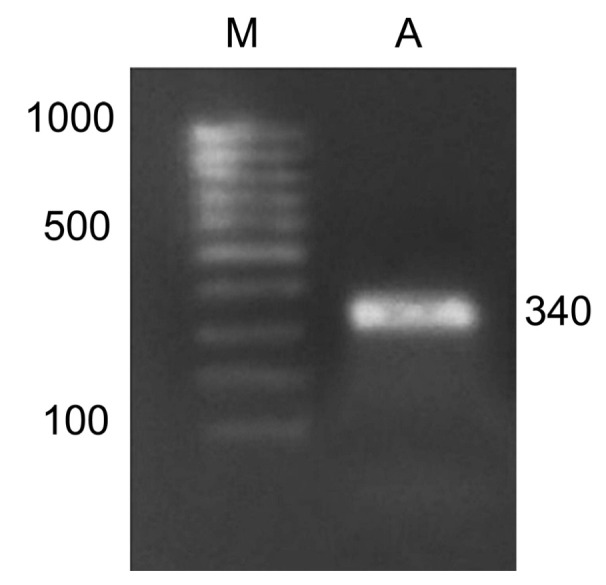
Amplification product of maize cDNA with primers for the *Csy1* gene encoding the mitochondrial from of citrate synthase. M, DNA length markers; A, the amplicon.

The study of adenine methylation patterns was performed on the *Csy1* gene promoter using methyl-specific restriction. For this, amplification of the modified DNA with primers to the promoter region of the citrate synthase gene was implemented, which made it possible to obtain products with the size of 340 nucleotides (Figure 6). For this, the following nucleotide sequences were used: forward, TAATGGGGGTTATGTGTATGTGTT; reverse, CAAATAAAAAATCCCATCAAATCAC. The resulting amplicons were used to assess the methyl status of the adenines in their GATC sites.

The nucleotide sequence of the amplicon contains three GATC sites that are the targets for the restriction endonuclease MboI. Cleavage at these sites makes it possible to obtain the restriction analysis products of 290, 254 and 121 nucleotides in size.

**Figure 7:**
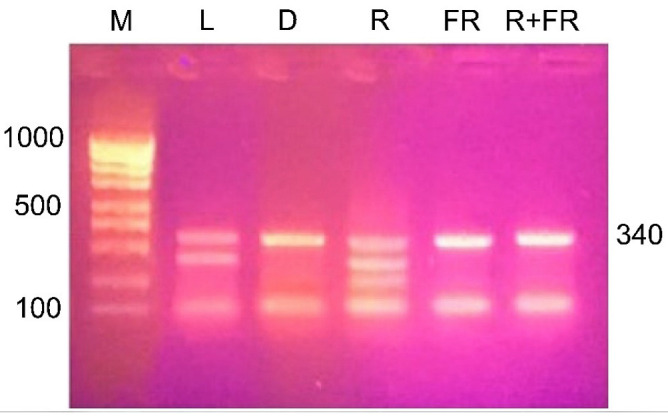
Restriction analysis of the nucleotide sequence with the restriction endonuclease MboI, specific for non-methylated adenine in the GATC site. M, DNA length markers; L, plants illuminated with white light; D, plants kept in darkness; R, plants illuminated with red (660 nm) light; FR, plants illuminated with far-red (730 nm) light; R+FR, plants illuminated with red (660 nm) followed by far-red (730 nm) light.

The results of restriction analysis of amplicons of the maize *Csy1* gene promoter under different light conditions indicate a change in its methyl status, as indicated by the difference in the amount of products of its specific cleavage by the MboI nuclease (Figure 7). Irradiation of plants with red light causes a change in the adenine methyl status of the analyzed amplicon, as evidenced by the presence of restriction products having size of 290, 254, and 121 nucleotides. When plants are exposed to red light, adenine methylation occurs at all three GATC sites present in the analyzed DNA sequence. A similar picture is observed in the white light variant, which shows the cleavage of the analyzed sequence with the formation of two products: 290 and 121 nucleotides. In this case, not all adenines of GATC sites are in the methylated state, ^254^A is not methylated.

The results of the restriction analysis of the samples from plants incubated in darkness or irradiated by far-red light (after darkness or after red light) indicate the uniformity of the state of the studied adenines. In these conditions, only one adenine at the position –121 is methylated, since only a single band is found on the electrophoregram after the treatment of DNA with the MboI nuclease.

## 3. Discussion

Substantial changes in the methyl status of the DNA of maize leaf cells under different light conditions associated with the modification of adenine have been observed in the experiments on methyl-specific restriction using the MAL1 endonuclease [35] (Figure 2 and Figure 7). An increase in the number of restriction products corresponds to an elevation in the proportion of methylated adenine, notably in the restriction sites for the MAL1 endonuclease (Figure 7). This restriction enzyme cleaves fully methylated sites only for a limited period of time; therefore, the ^6m^A sites in the maize genome identified by MAL1 are symmetrical and fully methylated [38]. The dependence of methylation on light regime demonstrates the phytochrome control of DNA adenine methylation [39,40]. The presence in the cell of the active form of phytochrome causes an increase in the methyl status of genomic DNA, due to the methyl-dependent modification of adenine. The opposite effect is caused by the action of far-red light and the successive action of red and far-red light (Figure 3). A similar dependence of DNA methyl status determined in the course of restriction analysis upon irradiation with far-red light and successive irradiation with red and far-red light indicates that phytochrome B is involved in the regulation of DNA adenine methylation, since phytochrome B is characterized by photoconversion, in contrast to phytochrome A [40,41]. Phytochrome B is the most common phytochrome of the type II (light stable), which requires a substantial fraction of P_fr_ to promote signaling in the low-fluence response [42]. Irrradiation by far-red light completely converts phytochrome B molecules to the form P_fr_ [43]. Phytochrome B null mutants demonstrate characteristic absence of reversible reactions red/far-red [44]. Contrary phytochrome A, which is light labile and controls a non-photoreversible reaction in response to irradiation of plants by low-fluence red light [45], phytochrome B is photoconvertible at low-fluence irradiations [40].

Phytochrome interacting factors, and in particular PIF4, act as central signaling elements in plant response to light [46]. The study of expression of the phytochrome B-interacting bHLH factor PIF4 in the plants incubated under white light or irradiated by red light showed a reduced level of its mRNA in comparison to the plants irradiated by far-red light (Figure 3). This suggests the suppression of this factor in response to the accumulation of the active form of phytochrome in the cell when plants are irradiated with red light. In this case, the activation of phytochrome acts as a negative regulator of *Pif4* gene expression in maize leaves. This means that PIF4 does not implement the phytochrome A-dependent signal, as the latter presumes a direct correlation between phytochrome A activation and PIF levels [40]. PIF4 selectively binds to the active form of phytochrome B and has a very low affinity to phytochrome A [36], while PIF1 and PIF3 interact with both phytochrome A and phytochrome B, thus PIF4 specifically acts as a regulator of the phytochrome B signaling pathway [47]. PIF4 functions as a negative regulator of phytochrome B signaling in Arabidopsis [47], where it exhibits a peak of transcript accumulation in leaves one day after dark treatment, which reveals its involvement in triggering dark-induced senescence [48,49]. Upon the irradiation by red light, phytochrome B is activated and then interacts with PIF4. The direct interaction between PIF4 and phytochrome B leads to the light-induced photophosphorylation followed by ubiquitination and PIF degradation [50,51].

Our results are consistent with the involvement of phytochrome B in the transduction of the light signal as its effect is related to photoconversion upon sequential exposure of plants to red and far-red light. The observed change in the level of *Pif4* gene transcripts indicates the operation of a negative regulatory mechanism characteristic for phytochrome B [47]. The obtained data demonstrate that the operation of Dam methylase is controlled by light via the phytochrome system, since its activity depends on the irradiation of plants with red and far-red light (Figure 4). Phytochrome B acts as a positive regulator of Dam methylase, and the decrease in PIF4 transcript level mediates this regulation mechanism. With the addition of a methyl group to the sixth position of the purine, the adenine ring can modify the interaction energy of bases and thus influence protein-DNA interactions [51]. It is possible that ^6m^A can regulate transcription by affecting transcription factors, RNA polymerases, histones, or other chromatin components. The specific correlation between ^6m^A and gene expression in different organisms is under regulatory control at different developmental stages [38,52]. The presence of ^6m^A in promoter regions causes the inhibition of gene expression, while its presence in the coding region correlates with the transcription activation, and thus adenine methylation in DNA acts as a regulatory mechanism in response to various stresses [53,54].

The study of the maize *Csy1* gene promoter showed the presence of adenine methylation sites (GATC) and E-box in its composition (Figure 5), which ensures interaction with transcription factors of the PIF family. In the dark, phytochrome B exists in a biologically inactive form P_r_, while phytochrome-inducible factors (PIFs) accumulate in the nucleus and regulate the expression of genes that inhibit photomorphogenesis. Under the action of red light, phytochrome B turns into the biologically active form P_fr_ and interacts with PIF4. Previously, it was found that the PIF4 transcription factor exhibits a stronger affinity for the E-box region [55]. In addition, miRNAs are involved in the process of DNA methylation. In particular, miR165 and miR166 are required for methylation of the *PHABULOSA (PHB)* gene in Arabidopsis [7].

Analysis of the nucleotide sequence of the promoter of the mitochondrial citrate synthase gene revealed the presence of an E-box in its composition, which makes it possible to regulate this gene through phytochrome B (Figure 5). This specific site of interaction with PIF4 is located in the immediate region of potential sites of adenylate methylation, which may play an important role in the mechanism for controlling the rate of its expression by changing the methyl status of the adenine promoter.

The use of the DNA nitrite conversion method made it possible to analyze the methyl status of individual adenines in the promoter region of the mitochondrial citrate synthase gene by methyl-specific restriction. The use of restriction endonuclease MboI, which specifically hydrolyzes DNA at the GATC site, if it contains methylated adenine, showed that irradiation of maize plants with light of different wavelengths causes a change in the methyl status of the analyzed adenines in the nucleotide sequence of the *Csy1* gene promoter. The appearance of the active form of phytochrome in the cell upon irradiation with red light causes an increase in the proportion of methylated adenines in the promoter of the citrate synthase gene, as evidenced by an increase in the number of restriction products after MboI treatment. The opposite effect takes place upon the irradiation of maize plants with far-red light. In these conditions, the amount of methyl-dependent restriction product decreased, which indicates a phytochrome-dependent mechanism for controlling the DNA adenylate methyl status. Phytochrome B can play an important role in this regulatory mechanism, since the photoconversion of this receptor upon sequential exposure of plants to red and far-red light undergoes photoconversion [40].

**Figure 8:**
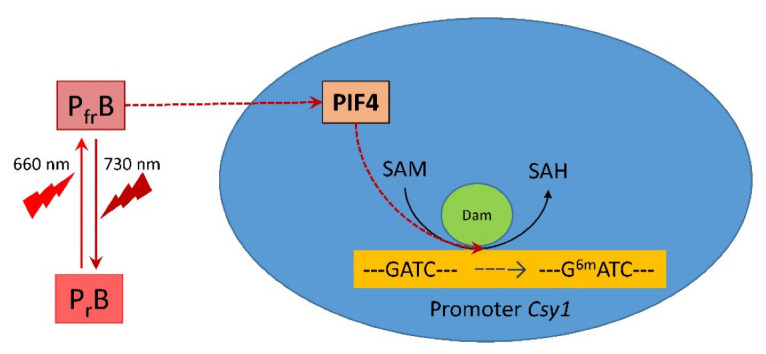
The mechanism of light regulation of the activity of mitochondrial citrate synthase in maize by phytochrome B via the decrease in expression of PIF4, causing a change in the adenylate methyl status of the *Csy1* gene promoter. Abbreviations: P_r_B, phytochrome B form sensitive to red (660 nm) light; P_fr_B, phytochrome B form sensitive to far-red (730 nm) light; PIF4, phytochrome interacting factor 4; Dam, DNA adenine methylase; *Csy1*, gene encoding mitochondrial citrate synthase; SAM, *S*-adenosyl-L-methionine; SAH, *S*-adenosyl-L-homocysteine; ^6m^A, *N*^6^-methyldeoxyadenine.

The obtained results on changes in the adenylate methyl status of the GATC sites of the *Csy1* gene promoter of the mitochondrial citrate synthase isoenzyme represent an epigenetic mechanism for regulating its transcription in maize leaves under changing light conditions. Modulation of the mitochondrial form of citrate synthase in the light may be one of the mechanisms of light-dependent regulation of plant respiration [28,56], in which the methyl status of the adenine promoter of the *Csy1* gene plays an important role. An increase in the proportion of methylated adenine in the GATC site decreases the efficiency of promoter interaction with the transcription factors, including PIF, which reduces the expression intensity of this gene. Figure 8 schematically presents a possible mechanism of the regulation of the mitochondrial citrate synthase by light via DNA adenine methylation mediated by phytochrome B.

## 4. Materials and Methods

The leaves of 14-day-old maize plants (*Zea mays* L., cv Voronezhskaya 76 obtained from the Voronezh branch of the All-Russian Research Institute of Maize), grown hydroponically in 12 h daylight of the intensity of 90 µmol quanta m^−2^ s^−1^, were used as an object for the study. White light was emitted by fluorescent lamps (growth setup Flora-1, PhytoSun, Moscow, Russia). Red and far-red light was obtained using LEDs with an emission region of 640–680 nm (KIPD40M40-K-P6, Kaskad-Elektro, Moscow, Russia) and 710–750 nm (ZL127A-5, Kaskad-Elektro, Moscow, Russia). The intensity of red or far-red light during irradiation was 4 μmoL quanta m^−2^ s^−1^, and the duration of irradiation was 15 min. This light intensity is sufficient for the occurrence of signal reactions associated with the participation of the phytochrome system but does not lead to the intensification of photosynthesis [32].

Genomic DNA from maize leaves was isolated by the phase distribution method based on phenol-chloroform extraction. Ammonium acetate (10 mM) was used as a specific precipitant [57]. The use of ammonium acetate to precipitate DNA during its isolation made it possible to increase its yield and purity [58]. Electrophoretic analysis of the resulting DNA preparation (Figure 1) indicated the absence of traces of impurities in the form of RNA. The MAL1 enzyme (SibEnzyme, Novosibirsk, Russia) was used to detect the methylated status of genomic DNA for adenine. The pattern of specific hydrolysis of genomic DNA from maize leaves under different light conditions was determined by the method of limited restriction analysis when processing 2 μg of DNA with 1 unit of the enzyme for 4 h at 37 °C [59].

The selection of primers for the *Pif4* gene was carried out using the Primer-BLAST program based on the mRNA nucleotide sequence (LOC100280260) taken from the NCBI database. Nucleotide sequences of the primers to the gene *Pif4* were as follows: the forward 5′-CCAAACTGGCTATTCGTCACT-3′, the reverse 5′-GGGTTCATTCTGAGGAAGAGA-3′. The reaction was performed at the following parameters: preliminary denaturing at 95 °C for 5 min, the cycle: 95 °C for 30 s, 58 °C for 40 s, 72 °C for 30 s (detection), 72 °C for 10 min (final elongation). The total RNA without the stage of reverse transcription was taken as a control.

For the isolation of Dam methylase [DNA adenine methylase; site-specific DNA-methyltransferase (adenine-specific), EC 2.1.1.72], a sample (1 g) was homogenized in a mortar with 5 mL of 50 mM Na-phosphate buffer (pH 7.5), containing 150 mM NaCl, and 1 mM dithiothreitol. The homogenate was centrifuged at 15,000 *×g* for 10 min at 4 °C; the resulting supernatant was used to determine the enzyme activity. The activity of Dam methylase was determined spectrophotometrically in 10 mM Tris-HCl buffer, pH 7.9, containing 50 mM NaCl, 10 mM MgCl_2_, 1 mM dithiothreitol, 80 µM *S*-adenosyl-L-methionine (SAM), and 40 nM DNA substrate [60]. All chemicals were from Sigma-Aldrich, St. Louis, MO, USA. The synthesized DNA substrate representing the sequence 3′-CAGGATCCATGCGATCAACCGATCAAGGATCCAC-5′ was used as a template for adenine methylation. The activity of the enzyme was monitored by the change in the optical density at 256 nm by the formation of *S*-adenosyl-L-homocysteine (SAH) in the reaction [61]. The unit of activity corresponded to the formation of 1 nmol of SAH per minute and was expressed per g of fresh weight (FW). All chemicals were from Sigma-Aldrich, St. Louis, MO, USA.

To perform nitrite-mediated DNA analysis, 0.7 µL acetic acid was added to a sample of 20 µL of DNA (100 µM), followed by the addition of 15 μL of freshly prepared 2 M sodium nitrite, thoroughly mixed, and incubated in a thermostat at 22 °C for 5 h [37]. The DNA was then re-precipitated from the reaction mixture with 10 M sodium acetate in the presence of ethanol and used for PCR. Amplicon restriction analysis for the non-methylated GATC site was performed using MboI endonuclease (Thermo Fisher Scientific, Waltham, MA, USA) according to the manufacturer’s recommendations.

The experiments were performed in three biological and four analytical repeats and the data were subjected to a two-way analysis of variance (ANOVA), employing general linear model for main effect ANOVA using STATISTICA version 9 data analysis software (Statsoft Wipro, East Brunswick, NJ, USA). Results are presented as mean ± standard deviation. The statistically significant differences at *p* < 0.05 were discussed. The images of electrophoregrams represent the data of a typical experiment repeated three to four times.

## 5. Conclusions

The results of this study demonstrate an increase in the proportion of methylated adenine, in particular in the restriction sites for the MAL1 endonuclease, which indicates a change in the methyl status of adenine in maize DNA depending on the light regime of plants. The obtained data show that the active form of phytochrome B exhibits an activating effect on Dam methylase. The use of DNA nitrite conversion followed by methyladenine-dependent restriction by MboI nuclease established a phytochrome B-dependent mechanism for changing the methyl status of adenine in the GATC site in the promoter of the mitochondrial citrate synthase gene *Csy1*. We conclude that the changes in the degree of adenine methylation of the promoter of the *Csy1* gene represent a new mechanism of the regulation of plant respiration by light. The future work on the role of adenine and cytosine DNA methylation in the regulation of plant photosynthetic and respiratory enzymes will further elucidate the operation of epigenetic mechanisms in plant adaptation to changing environment.

## Data Availability

The datasets generated for this study are available upon request from the corresponding author.

## Abbreviations

^6m^A	*N*^6^-methyl-2′-deoxyadenine
^5m^C	5-methyl-2′-deoxycytosine
CS	citrate synthase
Dam methylase or DNA adenine methyltransferase	site-specific DNA-methyltransferase (adenine-specific)
PIF	phytochrome interacting factor
SAM	*S*-adenosyl-L-methionine
SAH	*S*-adenosyl-L-homocysteine
TCA cycle	tricarboxylic acid cycle
